# Long-Term Physical, Cognitive, and Psychological Outcomes in Severe COVID-19 Patients Managed With Extracorporeal Membrane Oxygenation: A Prospective Study

**DOI:** 10.1097/MAT.0000000000001997

**Published:** 2023-07-29

**Authors:** Matteo Pozzi, Marco Giani, Mara Andreossi, Alice Annoni, Marta Villa, Valeria Bellin, Daniela Ferlicca, Simone Piva, Roberto Rona, Leonello Avalli, Alberto Lucchini, Giuseppe Foti

**Affiliations:** From the * Department of Emergency and Intensive Care, ASST Monza, Monza, Italy; † School of Medicine and Surgery, University of Milano-Bicocca, Monza, Italy; ‡ Department of Medical and Surgical Specialties, Radiological Sciences and Public Health, University of Brescia, Brescia, Italy; § Department of Anesthesia, Critical Care and Emergency, Spedali Civili University Hospital, Brescia, Italy.

**Keywords:** COVID-19, extracorporeal membrane oxygenation, adult respiratory distress syndrome

## Abstract

Extracorporeal membrane oxygenation (ECMO) has been used in highly selected COVID-19 patients with severe respiratory failure. Scarce data exist on long-term outcomes of these patients. We performed a single-center prospective evaluation of consecutive COVID-19 ECMO patients successfully discharged from the intensive care unit between February 2020 and January 2022. Physical, cognitive and psychological outcome was assessed at 3, 6, and 12 months by in-person evaluation. All the 34 discharged patients (median age 49 years old) were alive at one year, and 25 of them were evaluated at the follow-up clinic. 67% of patients had muscle weakness, with improvement over time (*p* = 0.032). The percentage of patients able to return to work progressively increased, up to 86% at 1 year. 23% of patients experienced fatigue. Participation restriction improved over time for both physical (*p* = 0.050) and emotional (*p* = 0.005) problems. Cognitive impairment, anxiety, and depression occurred in 29%, 29%, and 23% of patients, respectively, with no changes over time. Health-related quality of life was good. In conclusion, COVID-19 ECMO patients suffer from significant long-term sequelae. However, multidimensional outcomes continued to improve over the follow-up time.

Survivors from acute respiratory distress syndrome (ARDS) are often burdened by multifaceted and long-lasting sequelae. This new-onset physical, cognitive and psychological impairment after a critical illness is often referred to as “Post Intensive Care Syndrome” (PICS).^1^ Many of these patients cannot resume their previous work and experience a significant reduction in health-related quality of life (HRQoL).^2^ Moreover, they show an increased risk of rehospitalization and death, which is particularly high for ARDS survivors.^3^

Over the past two decades, the use of extracorporeal membrane oxygenation (ECMO) as a rescue therapy for the most severe ARDS patients has progressively increased. From February 2020, the huge number of COVID-19 patients with life-threatening respiratory failure led to a further increase in ECMO utilization. To date, more than 15,000 COVID-19 patients have been treated with ECMO worldwide, of which about half survived to hospital discharge.^4^ This constitutes a large cohort of patients at risk of long-term sequelae and in need of long-term follow-up care. Among ARDS patients, those managed with ECMO represent a particular subset characterized by higher severity, more invasive treatment, and more prolonged ICU length of stay (LOS). Although a clear association between clinical severity and long-term consequences has not been defined, it can be speculated that such a population may be at higher risk of PICS.

Little is known about the long-term consequences of ARDS due to COVID-19. It is unclear whether the characteristics and the severity of the long-term impairment in patients who suffered COVID-19 ARDS may differ from patients with ARDS from other etiologies. Moreover, data about long-term outcome of COVID-19 patients managed with ECMO may represent an important issue to improve patient selection and identify specific recovery-focused interventions.

This work aims to describe physical, psychological, and cognitive impairments—along with HRQoL—in a cohort of consecutive COVID-19 critically ill patients managed with ECMO and prospectively evaluated in-person at 3, 6, and 12 months after discharge.

## Materials and Methods

Data were gathered in the context of a larger multicentric prospective study on post-ICU follow-up (PICS Study) approved by the local Ethics Committee (Comitato Etico Brianza—NP3369). The study was registered at ClinicalTrial.gov (NCT: NCT04608994). Written informed consent was obtained from each participant at the time of the first follow-up visit.

This prospective observational study was conducted in a large academic hospital and ECMO center (San Gerardo Hospital, Monza, Italy) in the region of Lombardy, northern Italy. Since the first pandemic wave, Lombardy was the Italian region that admitted most ICU COVID-19 patients, and this huge number of critical care patients triggered a massive increase in ICU bed capacity.^5^ To face this surge, from February 20, 2020, to January 1, 2022, our institution increased several times its standard ICU capacity (33 beds), managing up to 90 COVID-19 critically ill patients simultaneously.

All consecutive adult COVID-19 patients treated with ECMO and successfully discharged from February 20, 2020, to January 1, 2022, were included in the present study. General criteria for ECMO candidacy followed current evidence and institutional protocols and are provided in Supplementary Material, Supplemental Digital Content 1, http://links.lww.com/ASAIO/B57.

This study was conducted and reported based on the Strengthening the Reporting of Observational Study in Epidemiology (STROBE) guidelines.

### Follow-Up Protocol and Measurements

The post-ICU follow-up program provides a multidimensional longitudinal evaluation of adult patients discharged from the ICU and includes clinical screening for psychological, cognitive, and physical sequelae.

A dedicated team, consisting of an ICU nurse and an ICU physician, provided a standard in-person evaluation at 3, 6, and 12 months after ICU discharge. The multidimensional patient evaluation included the following measures:

1. Nutritional status was evaluated by body mass index (BMI) and Mini Nutritional Assessment (MNA), with an MNA < 12 indicating malnutrition or malnutrition risk.^6^
2. Physical Function was assessed according to the International Classification of Functioning, Disability and Health (ICF) of the World Health Organization^7^ investigating physical performance in three different areas:
  - *Impairment at the body level*. Muscle weakness was evaluated using the Medical Research Council sum score (MRCss), which provides a global estimate of muscle strength ranging from 0 to 60. MRCss < 48 indicates significant reduction of muscular strength.^8^ Handgrip dynamometry of the dominant hand^9,10^ was obtained and normalized for age and sex.^11^ Values < 70% of predicted were considered as significantly reduced.
  - *Activity limitation* was assessed by the six-minute walking test (6MWT) performed according to recommendations.^12^ Walked distance was normalized to the predicted value.^13^ A qualitative evaluation of the ability to walk was performed by Functional Ambulation Scale (FAC). Self-reported fatigue was investigated by Fatigue Severity Score (FSS), and an FSS > 36 was considered as an indicator of significant fatigue.^14^ Self-reported dyspnea was quantified according to the Medical Research Congress Dyspnea Scale.^15^
  - *Participation restriction.* Role of physical and mental problems in preventing patients from social participation was measured by 36-Item Short-Form Health Survey (SF-36 Version 1) role limitation items.^16^ Scoring method is detailed below.

3. Mental and cognitive evaluation included several instruments:
  - *Cognitive function* was evaluated by Montreal Cognitive Assessment (MoCA), which explores different cognitive domains with MoCA score <26 indicating cognitive impairment.^17^
  - *Self-reported anxiety and depression* symptoms were assessed with Hospital Anxiety Depression Scale (HADS), which provide two subscore for anxiety (HADS-A) and depression (HADS-D), that are likely to be present with both subscores > 7.^18^
  - *Self-reported symptoms of PostTraumatic Stress Disorder (PTSD*) were investigated by the Posttraumatic Stress Disorder Checklist for DSM-5 (PCL-5). A PCL-5 > 32 is consistent with the presence of PTSD.^19^
  - *Sleep disturbances* were evaluated by Insomnia Severity Index (ISI). ISI > 8 was considered abnormal.^20^
4. HRQoL was investigated using SF-36 version 1,^16^ a validated HRQoL measure which cover eight dimensions: PF—physical functioning; RP—role limitations due to physical health problems; SF—social functioning; BP—bodily pain; MH—general mental health, covering psychological distress and well-being; RE—role limitations due to emotional problems; VT—vitality, energy, or fatigue; GH—general health perceptions. Items were scored as recommended,^21^ and two higher-order summary scores, the physical component summary (PCS) and the mental component summary (MCS) scores were calculated.^22^ For each item, a score above 50 was considered as normal.

Baseline characteristics (demographic, anthropometric, clinical severity data) were collected at the time of ICU admission. Ventilatory data refers to the time immediately prior to ECMO initiation.

To analyze the occurrence and the co-occurrence of relevant impairment in different area of physical functioning, patients were classified according to the presence of:

1. Muscle Weakness, defined by MRC < 48 or HGD < 70% of predicted value.
2. Activity Limitation, defined as the presence of 6MWT distance < 70% of predicted, FSS > 36, FAC < 5 (not independent walking) or the impossibility to resume work.
3. Participation restriction, defined as the presence of a SF-36 score for role limitation for emotional or physical problems < 50.

#### Statistical Analysis

Quantitative variables were reported using median and interquartile range (IQR), while count and percentage were used for categorical variables. A sample size calculation was not performed, since the study sample was represented by all eligible patients discharged during the study period. Differences among continuous variables were tested by an unpaired Student’s t-test or nonparametric Wilcoxon test for normally distributed variables. To compare continuous outcomes measured at 3, 6, and 12 months, a mixed-effect model was used. The follow-up time (3, 6, and 12 months) was included as a fixed effect, whereas patients were considered as random effect. To compare distribution of categorical outcome variables at 3, 6, and 12 months a cumulative link mixed model was used. No data imputation was provided for missing values. All tests were two-sided and a *p* value less than 0.05 was considered statistically significant. Statistical Analysis was performed with SPSS (SPSS Incorporation, Chicago, IL).

## Results

During the study period, a total of 50 patients with COVID-related ARDS were treated with ECMO. Sixteen patients died during ECMO support. The other 34, which were successfully weaned from ECMO and discharged from the ICU, were included in the present study.

Venovenous (V-V) ECMO was used in 48 patients (96%), whereas 2 patients required venoarterial (V-A) ECMO due to concomitant cardiogenic shock. The more common ECMO configuration was Femoro-Femoral (70%). The median age was 53 years (44–58) and 34 (68%) patients were males. Most patients were actively employed (76%), without comorbidities (56%), and with a Clinical Frailty Scale of 1 or 2 (60%), indicating a very fit/fit performance status. Table 1 resumes the demographic and clinical characteristics of the survivors, stratified according to follow-up attendance.

**Table 1.** Baseline Characteristics of the Study Population

	All Survivors (N = 34)	Patients With Follow-Up Evaluation (N° = 25)	Patients Without Follow-Up Evaluation (N° = 9)	*p*
Variables at ICU admission				
Age, years, median (IQR)	49 (42–56)	51 (38–55)	48 (46–57)	0.625
Sex male, N (%)	20 (58%)	16 (64%)	5 (44%)	0.310
Body mass index, kg/m^2^, median (IQR)	32 (27.3–36.2)	31.2 (27.1–35.9)	32.6 (31.4–37.3)	0.320
Employment status, employees, N (%)	26 (89%)	22 (88%)	4 (100%)	0.464
No. comorbidities, N (%)				0.346
0	22 (65%)	15 (60%)	7 (78%)	
1	6 (18%)	6 (24%)	0 (0%)	
2	5 (15%)	3 (12%)	2 (22%)	
3	1 (3%)	1 (4%)	0 (0%)	
>3	0 (0%)	0 (0%)	0 (0%)	
Charlson comorbidity index, median (IQR)	1 (0–2)	1 (0–2)	1 (0–1)	0.462
SAPS II score, median (IQR)	32 (25–39)	33 (27–39)	28 (21–37)	0.266
SOFA score, median (IQR)	6.5 (4–7)	7 (4.5–7)	5 (4–7)	0.619
Hospital admission to ICU admission, days, median (IQR)	3 (0–7)	3 (0–7)	4 (0–6)	0.362
Duration of NIMV before ICU admission, days, median (IQR)	1 (0–5)	3 (0–5)	1 (0–3)	0.151
Therapeutic interventions during ICU stay				
Inhaled nitric oxide, N (%)	3 (9%)	1 (4%)	2 (22%)	0.098
Prone position, N (%)				
Before ECMO	25 (73%)	20 (80%)	5 (55%)	0.154
During ECMO	26 (76%)	19 (76%)	7 (78%)	0.914
Any time	30 (88%)	23 (92%)	7 (78%)	0.256
Tracheostomy, N°(%)	11 (33%)	9 (36%)	2 (25%)	0.566
Continuous renal replacement therapy, N (%)				
Before ECMO	0 (0%)	(0%)	0 (0%)	–
During ECMO	3 (9%)	3 (12%)	0 (0%)	0.276
Any time	3 (9%)	3 (12%)	0 (0%)	0.276
Glucocorticoids, N (%)		21(84%)	4 (44%)	
COVID-19 protocol (dexamethasone)	15(44%)	12 (48%)	3 (75%)	0.536
ARDS protocol (methylprednisolone)	10(29%)	9 (36%)	1 (25%)	
Septic shock protocol (hydrocortisone)	4 (12%)	4 (16%)	0 (0%)	
Any	29 (85%)	25(100%)	4 (44%)	0.389
Vasopressors, N°(%)	15 (44%)	13 (52%)	2 (22%)	0.921
Tocilizumab, N°(%)	3 (9%)	3 (12%)	0 (%)	0.276
Mechanical ventilation, N (%)	34 (100%)	25 (100%)	9 (100%)	–
Duration of mechanical ventilation, days, median (IQR)	25 (18–46)	25 (19–50)	25 (15–32)	0.197
ICU admission to ICU discharge, days, median (IQR)	27 (20–45)	28 (21–49)	26 (16–36)	0.148
ECMO				
Symptoms onset to ECMO, days, median (IQR)	11 (8–14)	11 (8–16)	8 (2–10)	0.091
Hospital admission to ECMO, days, median (IQR)	6 (2–9)	7 (3–10)	6 (1–7)	0.217
ICU admission to ECMO, days, median (IQR)	1 (1–4)	2 (1–5)	1 (0–3)	0.227
Mechanical ventilation duration before ECMO, days, median (IQR)	1 (1–4)	2 (1–5)	1 (0–3)	0.227
ECMO duration, days, median (IQR)	12 (9–19)	14 (9–22)	10 (7–15)	0.101
ECMO type, N (%)				0.382
V-V ECMO	32 (94%)	23 (92%)	9 (100%)	
V-A ECMO	2 (6%)	2 (8%)	0 (0%)	
ECMO configuration				0.085
Femoral-Femoral	26 (76%)	21 (84%)	5 (56%)	
Femoral-Jugular	8 (23%)	4 (16%)	4 (44%)	
Bilumen Jugular cannula	0 (0)	0 (0%)	0 (0%)	
Major bleeding*, N (%)	6 (18%)	5 (20%)	1 (11%)	0.549
Intracranial bleeding, N (%)	0 (0)	(0%)	0 (0%)	-
Respiratory variables before ECMO initiation, median (IQR)				
Tidal volume, ml	400 (330–430)	400 (330–430)	360 (335–440)	0.938
pO_2_/FiO_2_ ratio, mmHg	70 (53–89)	69 (52–87)	82 (55–95)	0.402
Static compliance of respiratory system, mL/cmH_2_O	28 (24–36)	27 (23–36)	29 (21–38)	0.984
Respiratory rate, bpm	28 (23–30)	28 (22–30)	24 (21–29)	0.377
Positive end expiratory pressure, cmH_2_O	14 (12–14)	14 (12–14)	12 (11–14)	0.263
Driving pressure, cmH_2_O	12 (11–15)	12 (11–15)	12 (10–15)	0.529
pH	7.37 (7.30–7.41)	7.37 (7.32–7.41)	7.38 (7.30–7.44)	0.978
pCO_2_, mmHg	55 (47–61)	56 (48–61)	50 (43–58)	0.213
ICU outcome				
Destination after ICU discharge, N (%)				0.307
High dependency unit	26 (76%)	20 (80%)	6 (67%)	
Transferred to other ICU	2 (6%)	2 (8%)	0 (%)	
General ward	6 (18%)	3 (12%)	3 (33%)	
Ventilatory assistance at ICU discharge, N (%)				0.307
No ventilatory assistance	1 (3%)	1 (4%)	0 (0%)	
Noninvasive ventilation	15 (44%)	9 (36%)	6 (67%)	
Low-flow oxygen	10 (29%)	8 (32%)	2 (22%)	
Tracheostomy	8 (23%)	7 (28%)	1 (11%)	
Hospital outcome				
Hospital admission to hospital discharge, days, median (IQR)	53 (40–67)	52 (40–71)	54 (39–67)	0.984
ICU discharge to hospital discharge, days, median (IQR)	16.5 (12–21)	14 (11–20)	18 (15–27)	**0.042**
Destination after hospital discharge				
Home	15 (44%)	11 (44%)	4 (44%)	0.829
Subacute rehabilitation	19 (56%)	14 (56%)	5 (56%)	

Patients were stratified by follow-up program attendance (*i.e.*, follow-up evaluation available *vs*. patients lost at follow-up). Data, if not otherwise specified, are referred to the day of ICU admission.

* Major bleeding was defined as a bleeding event requiring transfusion of more than 10 ml/kg over a 24 hour period and/or requiring a surgical, endoscopy, or interventional radiology procedure.

ARDS, acute respiratory distress syndrome; ECMO, extracorporeal membrane oxygenation; ICU, intensive care unit; IQR, interquartile range; NIV, noninvasive ventilation; pCO_2_, arterial carbon dioxide tension; pO_2_/FiO_2_, ratio of arterial oxygen tension to inspiratory oxygen fraction; SAPS II, simplified acute physiology score II; SOFA, sequential organ failure assessment.

**Figure 1.**
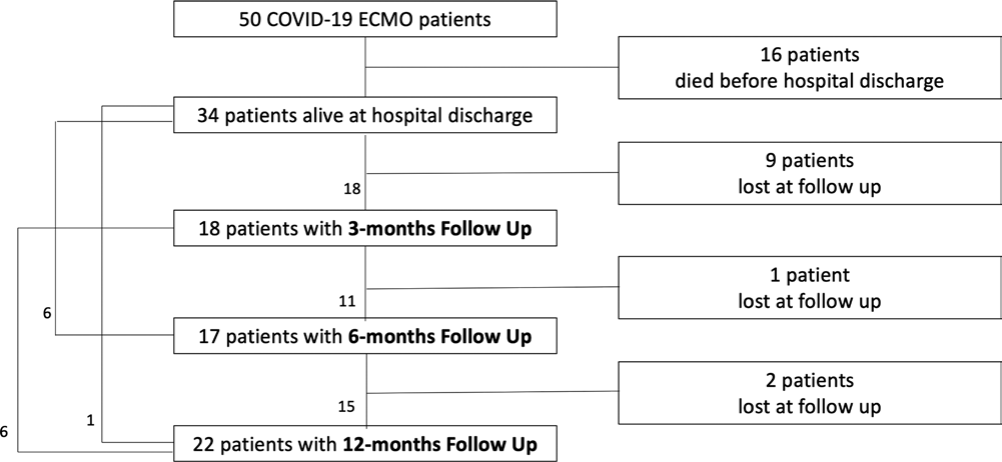
Study flow chart. Of 50 COVID-19 ECMO patients, 34 were successfully discharged from ICU, while 16 died while on extracorporeal membrane oxygenation (ECMO) support. All patients successfully weaned from ECMO were successfully discharge from hospital. Among survivors, 25 patients were evaluated at least once at follow-up clinic. Four patients were evaluated only once (1 patient at 3 months, 2 patients at 6 months and 1 patient at 12 months). Ten patients were evaluated twice (6 patients at 3 and 12 months and 4 patients at 6 and 12 months). Eleven patients were evaluated three times. ECMO, extracorporeal membrane oxygenation.

Twenty-five of 34 patients were evaluated at least once at the follow-up clinic and for 22 patient 12-month evaluation was available (Figure 1). Data regarding the 3- and 6-month evaluations are reported in Supplementary Material, Supplemental Digital Content 1, http://links.lww.com/ASAIO/B57 (Table S1, Supplemental Digital Content 1, http://links.lww.com/ASAIO/B57).

No patients died after ICU discharge throughout the follow-up period. Patients for whom follow-up data were available did not differ from patients who were lost to follow up for any demographic or clinical characteristic.

### Discharge Disposition and Ventilatory Assistance

Fifteen patients (44%) returned home after hospital discharge, while the remaining 19 (56%) were admitted to a subacute rehabilitation facility. All eight patients discharged from ICU with a tracheostomy were already weaned and decannulated at the time of first follow-up visit. No patient required oxygen support or any kind of ventilatory assistance at the time of the first follow-up visit.

### Nutritional Status

Patients lost weight after discharge but at 12 months regained the baseline BMI (BMI gain −0.3 (−5.6 to 0.9) kg/m^2^, Table 2) (Table S1, Supplemental Digital Content 1, http://links.lww.com/ASAIO/B57).

**Table 2.** Physical Outcome at 12 Months From Discharge

	12 Months (n = 22)
Time from ICU discharge to follow up, days, median (IQR)	381 (340–416)
Nutritional status	
Body mass index, kg/m^2^, median (IQR)	29.7 (25.2–34.5)
Body mass index gain, kg/m^2^, median (IQR)	−0.3 (−5.6 to 0.9)
Mini nutritional assessment score, median (IQR)	14 (13–14)
Risk of malnutrition/malnutrition, N (%)	0 (0%)
Physical function—muscle weakness	
MRC sum score, median (IQR)	60 (57–60)
Normal	16 (72%)
Mild weakness	1 (4%)
Significant weakness	1 (4%)
Severe weakness	2 (9%)
Significant weakness (MRC < 48), N (%)	3 (13%)
Handgrip dynamometry	
Handgrip dynamometry, kg, median (IQR)	32 (23–47.75)
Handgrip dynamometry, % of predicted, median (IQR)	98 (68.2–120)
Handgrip dynamometry < 70% of predicted, N (%)	5 (25%)
Foot drop, N°(%)	1 (4%)
Muscle weakness, N°(%)	7 (35%)
Physical function—activity limitation	
Return to work, N°(%)	
Yes	17 (77%)
Yes, with different tasks	2 (9%)
No	3 (14%)
Functional ambulation scale, N (%)	
Dependent on physical assistance	0 (0)
Dependent on supervision	2 (9%)
Independent-level surface only	1 (5%)
Independent	18 (86%)
Fatigue severity score	19 (10–50)
Fatigue severity score, median (IQR)	19 (10–50)
Fatigue severity score > 36, N (%)	6 (28%)
6MWT	
6MWT, m, median (IQR)	422 (392–495)
6MWT, percentage of predicted, median (IQR)	74.8 (62.8–82.6)
6MWT < 70% of predicted, N (%)	12 (63%)
MRC score for dyspnea, median (IQR)	0 (0–1)
Activity limitation, N (%)	14 (70%)
Physical function—participation restriction	
SF-36 role limitation due to physical problems	75 (12–100)
Significant role limitation due to physical problem (<50), n (%)	17 (77%)
SF-36 role limitation due to emotional problems	100 (50–100)
Significant role limitation due to emotional problem (<50), N (%)	14 (64%)
Participation restriction, N (%)	18 (81%)

6MWT, six-minute walking test; ECMO, extracorporeal membrane oxygenation; IQR, interquartile range; MRC score, Medical Research Congress score; SF-36, 36-Item Short-Form Health Survey.

### Muscle Weakness

When assessed by MRSss, median muscle strength was good (median MRCss 60 [57–60]) and only a small proportion of patients had significant weakness (13%), with no effect of follow-up time (Tables 2 and S1, Supplemental Digital Content 1, http://links.lww.com/ASAIO/B57).

Five (25%) patients had a significant impairment in handgrip strength (Table 2). This percentage, significantly improved over time (*p* = 0.032 for the percentage of impaired patients and *p* = 0.004 for handgrip strength values, see Table S1, Supplemental Digital Content 1, http://links.lww.com/ASAIO/B57).

At the end of follow-up period, only 1 (4%) patient showed an isolated deficit of foot dorsiflexion (MRC ≤ 1) with normal strength of the remaining lower limb muscle group, consistent with the diagnosis of common peroneal nerve (CPN) deficit.

### Activity Limitation

All patients lived at home at the time of the first follow-up evaluations. At 12 months, 17 (77%) patients had fully resumed their previous work (Table 2). This percentage significantly increase over time (*p* = 0.003, Table S1, Supplemental Digital Content 1, http://links.lww.com/ASAIO/B57).

Median FSS was 19 (10–50) and 6 (28%) patients experienced significant fatigue with no significant change during the follow-up period (Tables 2 and S1, Supplemental Digital Content 1, http://links.lww.com/ASAIO/B57). The median distance on 6MWT was 74.8% (62.8%–82.6%) of the predicted value, and the 6MWT was below 70% of predicted value in 12 (63%) patients, with no significant improvement over time. 18 (86%) patients were able to walk independently (Table 2) and this percentage remained stable with time (Table S1, Supplemental Digital Content 1, http://links.lww.com/ASAIO/B57).

### Participation Restriction

At 12 months, role limitation due to both physical and emotional problems was reported as significant in 17 (77%) and 14 (64%) patients (see Table 2). The prevalence of participation restriction reduced with time both for physical (p = 0.050) and emotional (p = 0.005) problems (Table S1, Supplemental Digital Content 1, http://links.lww.com/ASAIO/B57).

### Mental and Cognitive Impairment

Most patients had normal cognitive function, and the proportion of patients with a cognitive impairment (36% at the end of study period, Table 3) did not change over time (see Table S1, Supplemental Digital Content 1, http://links.lww.com/ASAIO/B57).

**Table 3.** Mental Outcome and Health-Related Quality of Life in Patients at 12 Months From Discharge

	12 Months (n = 22)
Mental and cognitive function	
Montreal cognitive assessment	
Montreal cognitive assessment, median (IQR)	27 (25−29)
Montreal cognitive assessment < 26, N (%)	8 (36%)
Hospital anxiety depression scale	
Anxiety score, median (IQR)	2 (0–4)
Anxiety score > 8, N (%)	1 (5%)
Depression score, median (IQR)	3 (1–6)
Depression score > 8, N (%)	3 (14%)
Posttraumatic stress symptoms checklist – 5	
Posttraumatic stress symptoms checklist – 5, median (IQR)	3 (1−8)
Posttraumatic stress symptoms checklist – 5 > 32, N (%)	1 (5%)
Insomnia severity index	
Insomnia severity index, median (IQR)	3 (1-6)
Insomnia severity index > 8, N (%)	3 (15%)
Mental and cognitive impairment, N (%)	9 (41%)
Health-related quality of life	
SF-36	
SF-36 physical health summary score	46 (34–52)
SF-36 mental health summary score	53 (29–57)
Physical health < 50, N (%)	13 (76%)
Mental health < 50, N (%)	7 (41%)

IQR, interquartile range; SF-36, 36-Item Short-Form Health Survey.

Significant anxiety and depression were reported by 1 (5%) and 3 (14%) patients (Table 3). There was a trend in reduction of anxiety prevalence throughout the follow-up period (Table S1, Supplemental Digital Content 1, http://links.lww.com/ASAIO/B57).

Only one patient reported PTSD symptoms (Table 3). The prevalence of PTSD did not change over time, although we could observe a trend in reduction in PCL-5 absolute value from 3 to 12 months (Table S1, Supplemental Digital Content 1, http://links.lww.com/ASAIO/B57).

Clinical overt insomnia was present in 3 (15%) patients, and this proportion did not improve during follow-up time (Tables 3 and S1, Supplemental Digital Content 1, http://links.lww.com/ASAIO/B57).

### Health-Related Quality of Life

PCS and MCS were below threshold for 13 (76%) and 7 (41%) patients, with no improvement over time (Table 3). Complete SF-36 results are provided in Table S2, Supplemental Digital Content 1, http://links.lww.com/ASAIO/B57.

### Co-occurrence of Physical Impairment

**Figure 2.**
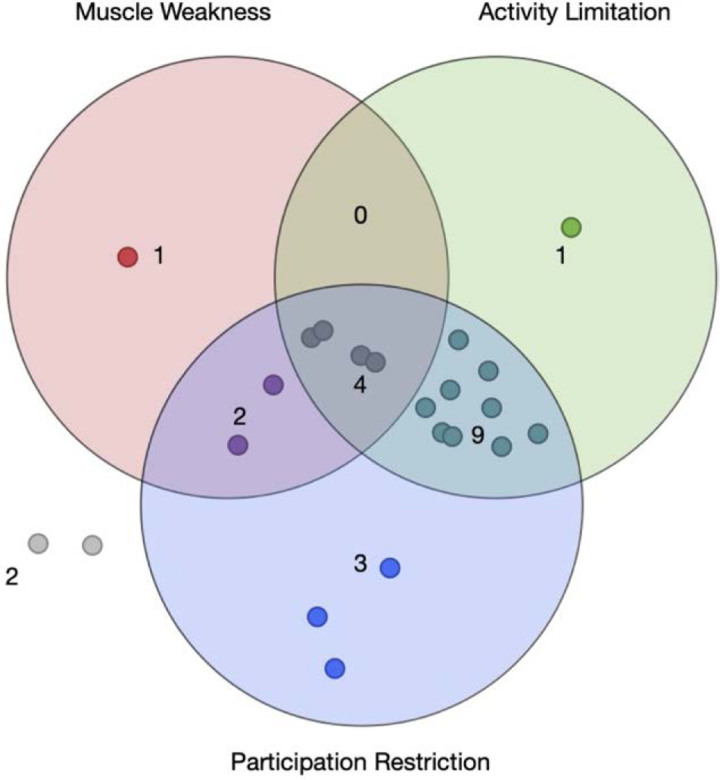
Co-occurrence of physical impairments. Venn diagram showing impairment in three different areas of physical function. Each point indicates one patient. Points enclosed into a circle represent a group of patients suffering from a specific physical impairment. Points enclosed in more than one circle represent patients suffering for more than one physical impairment. Fifteen patients had an impairment in almost two dimensions of physical functioning. All but three patients with participation restriction experienced activity limitation or muscle weakness. Only four patients showed an impairment in all these three dimensions of physical function. Only in one case muscle weakness occurred without other physical impairment.

Participation restriction was the most frequently impaired dimension of physical function (81% of patients), while the prevalence of muscle weakness and activity limitation was 35% and 70%, respectively. The prevalence of these three types of impairment did not significantly vary over the follow-up time. Co-occurrence of physical impairment is shown in Figure 2.

## Discussion

This prospective study describes long-term functional, psychological, and cognitive outcome of a cohort of consecutive COVID-19 patients managed with ECMO. Among patients who were successfully discharged from ICU, all were alive at 1 year and lived at home without any respiratory support. Eighty-nine percent of patients resumed previous work at 1 year. Many patients experienced muscle weakness after ICU discharge, but muscle strength recovered over time. The cognitive and psychological outcomes, such as quality of life, were relatively good and comparable to those reported for ARDS patients managed with or without ECMO.^23,24^

When critically ill patients are discharged from ICU, they start walking through a recovery trajectory. During this transition, their quality of life can be burdened by new and unresolving disabilities which affect several areas of physical and mental function.

Relatively limited information exists about long-term consequences of COVID-19, and therefore, COVID-19 survivors are anticipated to experience similar issues to those reported in prepandemic ARDS cohorts.^25^ In this context, the few studies which performed a direct comparison between these two populations did not demonstrate significant differences in terms of long-term outcome.^26–28^

Patients managed with ECMO represent a particular subset of highly selected ARDS patients, characterized by a greater clinical severity coupled with a young age. In previous studies, a significant number of ECMO survivors were reported to suffer from PICS.^23^ Available data lack a direct comparison between ECMO and non-ECMO patients to what extent long-term outcomes are associated with ECMO itself, even if the prevalence of long-term cognitive and psychological sequelae was similar to other survivors of critical illnesses.^23^

Many ICU survivors are unable to resume previous work. This may represent an additional burden of economic and social distress, thus increasing the impact of newly acquired disabilities.^29^ We found that return to work progressively increased during the first year from ICU discharge (up to 86% of patients). This result appears quite encouraging. The use of employment status as a pragmatic patient-centered endpoint could capture in a comprehensive way the improvement of many different and interplaying aspects of PICS syndrome, including ultimate social and economic consequences on patients and their families.^30^

Physical function can be impaired at different levels in ICU survivors: muscle weakness, fatigue, pain, limitation in daily activity and social participation. In particular, muscle weakness is a common consequence of critical illness that can persist for months after recovery.^31^ Moreover, strength reduction can be associated with ICU-acquired neuromuscular disorders, such as critical illness polyneuropathy and muscle atrophy, that represent specific disorders whose diagnosis requires electrophysiological investigations.^32,33^ Recent literature suggested the hypothesis that neuromuscular alterations, and hence the reduction of muscle force-generation capacity, could be one of the main drivers of the multifaceted functional impairment.

In our study, physical dysfunction was the most frequent consequence, and most patients presented impairment in two or more physical functioning areas. This could be consistent with the complex interplay between muscle strength, functional capacity, and social incorporation, even if the small sample size prevents us from any further consideration. However, it is worth noting as, while patients progressively return to work during the follow-up period, the only variable that showed a parallel statistically significant increase was handgrip dynamometry, which is an objective measure of muscle strength. These results are not directly comparable with those of previous studies on ECMO patients, which were mainly based on self-reported muscle assessment and lack of direct strength measurement^34–37^ (Table S3, Supplemental Digital Content 1, http://links.lww.com/ASAIO/B57).

Our results are consistent with those of Latronico et al,^38^ who recently applied the same methodological approach into a larger COVID-19 ARDS patient population, reporting MRCss HGD absolute value comparable to our results. HGD was reduced below normal values in 70% of patients (versus 67% in our cohort), and HGD was the only measure that improved with time.

A particular type of localized muscle weakness is the so-called Foot Drop phenomenon, which represents the inability to dorsiflex and evert the foot at the ankle, while the strength of remaining lower limb muscles is normal. It results from ipsilateral injury of Common Peroneal Nerve (CPN) [Citation error] and has been recently described as relatively frequent (around 8%) in a population of ECMO survivors.^39^

We found a relatively high prevalence of foot drop phenomenon (22% at three months), which was associated with significant functional limitation and global strength reduction but improved during time. Differently from previous studies, in which foot drop was mainly associated with ipsilateral cannulation for VA-ECMO, all our affected patients were on femoral V-V ECMO. Although mechanical damage is frequently recognized as a cause of CPN palsy, other mechanisms, relying upon the dependence of CPN upon a marginal blood supply, have been proposed.^40,41^

These results suggest the importance to recognize, for a research and clinical purpose, this specific neuromuscular deficit, along with its prevalence and mechanism, in ECMO survivors.

Prevalence of cognitive impairment was relatively low (29%) and consistent with previous reports in ECMO^23^ and non-ECMO ARDS population^24^ (Table S2, Supplemental Digital Content 1, http://links.lww.com/ASAIO/B57). Moreover, cognitive sequelae in ECMO patients may recognize specific causative mechanisms related to ECMO itself that are not involved in general ICU populations.^42^

The prevalence of anxiety, depression, and PTSD was low and quality of life was good. Our results are consistent with previous studies on classical ARDS patients treated^23^ or not^24^ with ECMO, whereas studies on COVID-19 ECMO population reported a wide range of anxiety prevalence, from 10 to 40%.^34,36^

The interaction between physical function and psychological factors play a complex and mostly not fully elucidated role in patient outcomes. Consistently to previous studies, we observed a trend toward improvement of psychological symptoms over time (i.e., reduction in anxiety prevalence and PCL-5 score), and this was parallel to the improvement in physical performance (i.e. recovery in muscle strength and improvement in activity limitation and participation restriction).

This is the first report that provides in-person evaluation of multidimensional long-term outcome in COVID-19 patients treated with ECMO. The in-person assessment allowed objective measurement of muscle strength and functional capacity, which represents the main strength of our work.

Although the sample size is relatively limited, our population is one of the largest reported to date (Table S3, Supplemental Digital Content 1, http://links.lww.com/ASAIO/B57).

Our work has also some limitations which deserve comment.

First, the study population is limited and consists of a highly selected group of young, physically fit, and generally healthy patients, with relatively low SOFA and SAPS II scores. If the number of patients whose outcome is described is at least similar to the previously reported COVID-19 ECMO populations,^34–37^ patients characteristics are consistent with the actual recommendation for ECMO candidacy in COVID-19 ARDS.^4^ Moreover, we did not perform a direct comparison between COVID-19 ARDS and ARDS from other etiologies. This actually limits the generalizability of our results. However, it also confirmed that satisfactory long-term outcomes can be achieved after extracorporeal support.

Second, we were not able to evaluate all discharged patients, since nine patients were lost to follow up. To reduce the risk of a selection bias, we ensured that these patients did not differ from the ones who were evaluated in term of illness severity, treatments and demographic characteristics. It is worth noting that this problem affects most of long-term investigations based on multiple *in-person* evaluations, and our rate of nonresponding patients was relatively low and comparable to previous works.^38^

Third, we did not achieve an exact 3- 6- and 12-month follow-up interval for all patients. In the same way, we were not able to obtain all the three scheduled evaluations for all the included patients. Lack of these data certainly represents a main limitation of this work and was due to restriction to hospital access and unwillingness of patients to attend the follow-up clinic during the subsequent pandemic waves which burdened our country. This may have limited the possibility of evaluating the recovery trajectory of each patient, and limited the capability of drawing definitive conclusions about improvement over time.

## Conclusions

COVID-19 patients who required ECMO suffer from significant long-term sequelae but we could observe an improvement in physical impairment during time. Among the more pragmatic patient-centered outcomes, at the end of the study period, 77% of patients were able to resume previous work. Further studies are required to confirm these findings in a larger population of ARDS patients supported with ECMO.

## Acknowledgments

Investigators of the Monza Follow-Up Study Group: Francesca Bettini, Diego Boaretto, Alfio Bronco, Mariangela Calabria, Mara Clementi, Stefano Gatti, Fabrizia Mauri, Valeria Meroni, Simone Sosio, Alessandra Valentino, Veronica Vigo.

## Supplementary Material


