# Supplementary material for: Long-Term Physical, Cognitive, and Psychological Outcomes in Severe COVID-19 Patients Managed With Extracorporeal Membrane Oxygenation: A Prospective Study
Source: ASAIO J. 2023 Jul 29;69(8):e376–83. doi: 10.1097/MAT.0000000000001997 (PMC10627404; doi:10.1097/MAT.0000000000001997)
Supplement: Supplementary file 1 [file mat-69-e376-s001.pdf]

Long-term physical, cognitive and psychological outcome in severe COVID-19 patients managed with Extracorporeal Membrane Oxygenation: a prospective study.

Supplementary Material

Matteo Pozzi*, Marco Giani, Mara Andreossi, Alice Annoni, Marta Villa, Valeria Bellin, Daniela Ferlicca, Simone Piva, Roberto Rona, Leonello Avalli, Alberto Lucchini, Giuseppe Foti and the Monza Follow-Up Study Group

Criteria for ECMO candidacy of patients with COVID-19 related ARDS.

1. age below 70 years
2. Absence of multiple high risk comorbidity (diabetes mellitus, obesity, arterial hypertension, chronic cardiac disease), severe immunosuppression or advanced malignancy.
3. less than 7 days of mechanical ventilation before ECMO implementation
4. P/F ratio < 50 for > 3 hours or a P/F ratio < 80 for > 6 hours despite
    a. optimization of mechanical ventilation (FiO2 > 80%, PEEP > 10, VT 6 ml/Kg)
    b. use of adjunctive therapies for ARDS (prone positioning, paralysis, recruitment maneuver, iNO)

    OR arterial pH < 7.25 with paCO2 > 60 mmHg for > 6 hours despite

    c. Respiratory Rate 35 bpm
    d. Ventilatory setting adjusted to keep Pplat < 32 mmHg

Table S1. Physical, mental and psychological outcome at 3, 6 and 12 months from discharge.

	3 Months (n = 18)	6 Months (n = 17)	12 Months (n = 22)	p[a]
Time from ICU Discharge to Follow Up, days, median (IQR)	96 (78-128)	224 (141-257)	381 (340-416)	< 0.001
**Nutritional Status**				
Body Mass Index, Kg/m2, median (IQR)	28.2 (25.6-31.5)	29.4 (26.4-33.3)	29,7 (25.2-34.5)	0.099
Body Mass Index gain, Kg/m2, median (IQR)	-2.6 (-5.4 – 0)	-3.4 (-7.6- -0.7)	-0.3 (-5.6 – 0.9)	0.014[b]
Mini Nutritional Assessment Score, median (IQR)	11 (9-12)	14 (13-14)	14 (13-14)	< 0.001[c]
Risk of Malnutrition/Malnutrition, N°(%)	3 (16%)	1 (6%)	0 (0%)	0.012
**Physical Function – Muscle Weakness**				
MRC Sum Score, median (IQR)	56 (47-60)	59 (55-60)	60 (57-60)	0.238
Normal	10 (56%)	13 (76%)	16 (72%))	0.286
Mild Weakness	4 (22%)	3 (18%)	1 (4%)	
Significant Weakness	3 (17%)	1 (6%)	1 (4%)	
Severe Weakness	1 (5%)	0 (0)	2 (9%)	
Significant Weakness (MRC < 48), N°(%)	4 (22%)	1 (6%)	3 (13%)	0.198
Handgrip Dynamometry				

Handgrip Dynamometry, kg, median (IQR)	19.5 (14-31.5)	28 (16.5-32)	32 (23-47.75)	0.001[d]
Handgrip Dynamometry, % of predicted, median (IQR)	59 (37.8-84.7)	73.7 (40.4-96)	98 (68.2-120)	0.004[e]
Handgrip Dynamometry < 70% of predicted, N°(%)	12 (67%)	7 (41%)	5 (25%)	0.032
Foot Drop, N°(%)	4 (22%)	1 (6%)	1 (4%)	
Muscle Weakness, N°(%)	12 (71%)	7 (41%)	7 (35%)	0.072
**Physical Function – Activity Limitation**				
Return to work, N°(%)				
Yes	4 (23%)	9 (53%)	17 (77%)	0.003
Yes, with different task	5 (27%)	1 (12%)	2 (9%)	
No	9 (50%)	6 (35%)	3 (14%)	
Functional Ambulation Scale, N°(%)				
Dependent on physical assistance	1 (5%)	0 (0)	0 (0)	0.470
Dependent on supervision	1 (5%)	1 (6%)	2 (9%)	
Independent level surface only	4 (22%)	3 (18%)	1 (5%)	
Independent	12 (67%)	13 (76%)	18 (86%)	

Fatigue Severity Score	27 (17-40)	21 (14-45)	19 (10-50)	
Fatigue Severity Score, median (IQR)	27 (17-40)	21 (14-45)	19 (10-50)	0.679
Fatigue Severity Score > 36, N°(%)	4 (23%)	4 (25%)	6 (28%)	0.935
6MWT				
6MWT, m, median (IQR)	391 (230-520)	422 (382-450)	422 (392-495)	0.249
6MWT, percentage of predicted, median (IQR)	71.4 (34.3-94.8)	74.3 (66.9-81.4)	74.8 (62.8-82.6)	0.766
6MWT < 70% of predicted, N°(%)	9 (50%)	12 (70%)	12 (63%)	
MMRC Score for dyspnea, median (IQR)	1 (0-2)	0 (0-1)	0 (0-1)	0.760
Activity Limitation, N°(%)	13 (72%)	12 (70%)	14 (70%)	0.823
**Physical Function – Participation Restriction**				
SF-36 Role limitation due to physical problems		50 (0-100)	75 (12-100)	0.068
Significant role limitation due to physical problem (< 50), n(%)		15 (88%)	17 (77%)	0.049
SF-36 Role limitation due to emotional problems		100 (33-100)	100 (50-100)	0.081
Significant role limitation due to emotional problem (< 50), N°(%)		14 (82%)	14 (64%)	0.005

Participation restriction, N°(%)		15 (88%)	18 (81%)	
**Mental and Cognitive Function**				
Montreal Cognitive Assessment				
Montreal Cognitive Assessment, median (IQR)	27 (25-28)	28 (26-29)	27 (25-29)	0.591
Montreal Cognitive Assessment < 26, N°(%)	5 (29%)	3 (18%)	8 (36%)	0.322
Hospital Anxiety Depression Scale				
Anxiety Score, median (IQR)	2 (0-8)	3 (1-8)	2 (0-4)	0.301
Anxiety Score > 8, N°(%)	5 (29%)	4 (23%)	1 (5%)	0.084
Depression Score, median (IQR)	3 (0-7)	6 (1-8)	3 (1-6)	0.088
Depression Score > 8, N°(%)	4 (23%)	5 (31%)	3 (14%)	0.460
Post-traumatic Stress Symptoms Checklist - 5				
Post-traumatic Stress Symptoms Checklist – 5, median (IQR)	7 (2-19)	5 (1-19)	3 (1-8)	0.061
Post-traumatic Stress Symptoms Checklist – 5 > 32, N°(%)	1 (14%)	3 (19%)	1 (5%)	0.409

Insomnia Severity Index				
Insomnia Severity Index, median (IQR)	4 (0-7)	3 (0-5)	3 (1-6)	0.604
Insomnia Severity Index > 8, N°(%)	2 (18%)	2 (12%)	3 (15%)	0.921
Mental and Cognitive Impairment, N°(%)	9 (50%)	9 (53%)	9 (41%)	0.729

IQR: Interquartile Range. ECMO: Extracorporeal Membrane Oxygenation. 6MWT: Six Minutes Walking Test.

a) If not otherwise specified, p values refer to a mixed effect model in which follow up time (3, 6 and 12 months) was defined as a fixed effect and patients were considered as random effect.

b) P value refers to a mixed effect model for the difference between BMI gain (i.e the difference between actual BMI and baseline BMI) from 3 to 12 months. In post-hoc analysis median BMI variation was significant at 6 months in comparison to 3 months ($p = 0.037$).

c) In post-hoc analysis median MNA at 12 months ($p < 0.001$) and at 6 months ($p = 0.001$) was significantly higher in comparison to 3 months.

d) In post-hoc analysis Handgrip Dynamometry, considered as an absolute value, at 12 months was significantly higher in comparison to 6 months ($p = 0.034$) and 3 months ($p = 0.001$).

e) In post-hoc analysis Handgrip Dynamometry, considered as percentage of predicted value, at 12 months was significantly higher in comparison to 6 months ($p = 0.014$) and 3 months ($p = 0.001$).

Table S2. 36-Item Short-Form Health Survey results at 6 and 12 months from discharge.

	**6 Months (n = 17)**	**12 Months (n = 22)**	p[a]
36-Item Short-Form Health Survey			
SF-36 Physical Functioning	60 (30-90)	80 (52-95)	0.405
SF-36 Role limitation due to physical problems	50 (0-100)	75 (12-100)	0.384
SF-36 Role limitation due to emotional problems	100 (33-100)	100 (50-100)	0.714
SF-36 Energy/Fatigue	75 (40-90)	75 (37-80)	0.419
SF-36 Social Functioning	75 (50-100)	87 (50-100)	0.680
SF-36 Emotional Well-being	88 (68-96)	76 (58-88)	0.1944
SF-36 Pain	67 (45-100)	67 (47-100)	0.606
SF-36 General Health	70 (45-85)	50 (32-80)	**0.0155**
SF-36 Physical Health Summary Score	41(30-53)	46 (34-52)	0.331
SF-36 Mental Health Summary Score	54(28-59)	53 (29-57)	0.478
Physical Health < 50, N°(%)	10 (67%)	13 (76%)	0.182
Mental Health < 50, N°(%)	7 (47%)	7 (41%)	0.288

a) p values refer to a mixed effect model in which follow up time (6 and 12 months) was defined as a fixed effect and patients were considered as random effect.

Table S3. Selected studies reporting long term outcome in COVID-19 and classical Acute Respiratory Distress Syndrome (ARDS) patients treated with Extracorporeal Membrane Oxygenation (ECMO).

		Selected studies on COVID-19 ECMO patients				Selected studies on Classical ARDS ECMO patients											
	**Present study**	**Taylor LJ** at al.[1]	**Gribens k A** et al.[2]	**Smith DE** et al [3]	**Rajajee** et al.[4]	**Holzgraefe** et al[5]	**Sylvestre** et al[6]	**Schmidt** et al [7]	**Luyt** et al[8]	**Sanfilipp o** et al[9]	**Hodgso n** et al[10]	**Mirabel** et al[11]	**Muller** et al[12]	**Corsi** et al[13]	**Risnes** et al[14]	**Tramm** et al[15]	**Chen** et al[16]
**N°[1]**	22	15	21	27	14	7	22	67	12	33	15	28	41	7	28	24	32
**FU time (months)**	12	4	8	10	12	38	20	17	12	24	8	17	32	19	60	12	12
**FU protocol**	In person	In person	Remote	In person	Remote	In person	Remote	Remote	In person	Remote	Remote	mixed	Remote	Remote	In person	Remot e	Remote
**Age (years)**	51	48	41	42	41	31	41	37	35	41	35	38	53	51	38	42	52
**Sex, male (%)**	64	59%	63%	80%	57%	71%	55%	55%	42%	73%	48%	43%	85%	35%	53%	75%	84%
**BMI (kg/m2)**	31	33.8	35.6	30	34	-	28	29	30	-	32	23	25	29	-	-	27
**pO_2/FiO_2**	69	76	64	80	94	51	-	53	73	57	69	-	-	-	-	-	-
**ECMO duration (days)**	14	11	9	19	16	23	12	15	9	19	11	10	8	3	15 (VV) 6 (VA)	6	8
**ECMO type**	VA/VV	VV	VV	VV	VA/VV	VV	VV	VA/VV	VV	VV	VV	VA	VA	VA	VA/VV	VA/VV	VA/VV
**MV duration (days)**	25	28	23	38	43	-	36	40	36	33	15	-	-	13	-	7	15
**ICU Length of Stay**	28	37	35	40	51	-	46	-	37	-	21	26	21	17	-	16	17

**Bleeding (%)**	20%	40.6%[2]	8%[3]	40%[3]	-	-	-	44%	-	-	-	-	9%	-	35%-	42%	-
**Discharge disposition (%)**	H: 54%<br>R: 56%	H: 39 %<br>R: 44%	H: 29%<br>R: 46%	H: 15%<br>R: 85%	H 29%<br>R 7%	-	-	-	-	-	H: 44%<br>R: 6%	-	-	-	-	-	-
**Alive at FU time (%)**	100%	100%	100%	96%	100%	-	91%	93%	-	86%	94%	96%	87%	-	93%	100%	91%
**Living at home at FU time (%)**	100%	100%	-	97%	92%			-	100%			-	-			-	-
**Back to work at FU time (%)**	86%	20%	-	4%	50%			52%	83%		55%)	50%	-	28%	50%	69%	-
**Indipendent subjects at FU time (%)**	100%	-	43%	-	92%	-	-	-	-	-	-	-	-	71%	21%	-	-
**6MWT (m or % of patient with an abnormal result)**	63%	100%	-	350	-	-	-	-	-	-	-	-	-	-	-	-	-
**Cognitive impairment (%)**	36%	46%	-	-	0%	14%	55%	-	-		-	-	-		43%	48%	
**Depression (%)**	14%	10%	-	-	60%	-	36%	25%	28%	42	-	12%	20%	43%	-	8%	13%
**Anxiety (%)**	5%	10%	-	-	40%	-	55%	34%	50%	42	-	38%	34%	28%	-	21%	-
**PTSD (%)**	5%	10%	-	-	40%	-	33%	16%	41%	27	-	27%	5%	28%	-	12%	-
**Fatigue (%)**	28%		62%	-	-	-		-	-					-	-	-	-

**HRQoL physical component**	46	-	42	-	-	-	36	45	-	42	-	-	50	-	-	45	49
**HRQoL mental component**	53	-	51	-	-		45	50	-	52	36	-	50	-	-	50	58
**Ventilatory Assistance (%)**	0%	18%	5%	4%[4]	15%[5]	-	-	-	-	-	-	-	-	-	-	-	-

.

(1) N° refers to the size of the population with follow-up data available. (2) Any bleeding event; (3) bleeding event requiring an intervention; (4) 1/27 patient required low flow oxygen; (5) One patient remained on mechanical ventilation at a long term facility and another one needed long term low flow oxygen

FU: Follow-up; BMI: Body Mass Index; ECMO: Extracorporeal Membrane Oxygenation; MV: Mechanical Ventilation; ICU: Intensive Care Unit; 6MWT: Six Minutes Walking Test; PTDS: Post-traumatic Stress Disorder; HRQoL: Health-Related Quality of Life.

Table represents a visual comparison of the results of the present work (first column) with those of the more recent studies, without any systematicity purpose. The reported studies have been chosen by searching Pubmed for papers on physical, cognitive and psychological outcome in ARDS patients managed with ECMO with or without COVID-19 etiology and published in the last 15 years.

Details on study design were gathered by each paper. “In person” follow-up refers to study providing a fully *in person* evaluation at a follow-up clinic, while “remote” follow-up refers to other forms of patient investigation, such as phone calls. “Ventilatory assistance” refers to any kind of respiratory device from low-flow oxygen to invasive mechanical ventilation. Numbers in brackets indicate the references. Diagnostic instruments and thresholds vary among the studies and so results could not be directly comparable.

References

1. Taylor, L.J.; Jolley, S.E.; Ramani, C.; Mayer, K.P.; Etchill, E.W.; Mart, M.F.; Fakhri, S.; Peterson, S.; Colborn, K.; Sevin, C.M.; et al. Early Posthospitalization Recovery after Extracorporeal Membrane Oxygenation in Survivors of COVID-19. *J. Thorac. Cardiovasc. Surg.* **2022**, doi:10.1016/j.jtcvs.2021.11.099.
2. Gribensk, A.; Schneider, A.; Gallaher, J.R.; Reid, T.S.; Kindell, D.G.; Charles, A.G.; Raff, L.A. Posthospitalization Outcomes after Extracorporeal Membrane Oxygenation (ECMO) for COVID-19. *Surgery* **2022**, *172*, 466–469.
3. Smith, D.E.; Chang, S.H.; Geraci, T.C.; James, L.; Kon, Z.N.; Carillo, J.A.; Alimi, M.; Williams, D.; Scheinerman, J.A.; Cerfolio, R.J.; et al. One-Year Outcomes With Venovenous Extracorporeal Membrane Oxygenation Support for Severe COVID-19. *Ann. Thorac. Surg.* **2022**, *114*, 70–75.
4. Rajajee, V.; Fung, C.M.-C.; Seagly, K.S.; Park, P.K.; Raghavendran, K.; Machado-Aranda, D.A.; Scott, J.W.; Delano, M.J.; Abou El Ela, A.S.A.A.; Haft, J.W.; et al. One-Year Functional, Cognitive, and Psychological Outcomes Following the Use of Extracorporeal Membrane Oxygenation in Coronavirus Disease 2019: A Prospective Study. *Crit Care Explor* **2021**, *3*, e0537.
5. Holzgraefe, B.; Andersson, C.; Kalzén, H.; von Bahr, V.; Mosskin, M.; Larsson, E.-M.; Palmér, K.; Frenckner, B.; Larsson, A. Does Permissive Hypoxaemia during Extracorporeal Membrane Oxygenation Cause Long-Term Neurological Impairment?: A Study in Patients with H1N1-Induced Severe Respiratory Failure. *Eur. J. Anaesthesiol.* **2017**, *34*, 98–103.
6. Sylvestre, A.; Adda, M.; Maltese, F.; Lannelongue, A.; Daviet, F.; Parzy, G.; Coiffard, B.; Roch, A.; Loundou, A.; Baumstarck, K.; et al. Long-Term Neurocognitive Outcome Is Not Worsened by of the Use of Venovenous ECMO in Severe ARDS Patients. *Ann. Intensive Care* **2019**, *9*, 82.
7. Schmidt, M.; Zogheib, E.; Rozé, H.; Repesse, X.; Lebreton, G.; Luyt, C.-E.; Trouillet, J.-L.; Bréchot, N.; Nieszkowska, A.; Dupont, H.; et al. The PRESERVE Mortality Risk Score and Analysis of Long-Term Outcomes after Extracorporeal Membrane Oxygenation for Severe Acute Respiratory Distress Syndrome. *Intensive Care Med.* **2013**, *39*, 1704–1713.
8. Luyt, C.-E.; Combes, A.; Becquemin, M.-H.; Beigelman-Aubry, C.; Hatem, S.; Brun, A.-L.; Zraik, N.; Carrat, F.; Grenier, P.A.; Richard, J.-C.M.; et al. Long-Term Outcomes of Pandemic 2009 Influenza A(H1N1)-Associated Severe ARDS. *Chest* **2012**, *142*, 583–592.
9. Sanfilippo, F.; Ippolito, M.; Santonocito, C.; Martucci, G.; Carollo, T.; Bertani, A.; Vitulo, P.; Pilato, M.; Panarello, G.; Giarratano, A.; et al.

Long-Term Functional and Psychological Recovery in a Population of Acute Respiratory Distress Syndrome Patients Treated with VV-ECMO and in Their Caregivers. *Minerva Anestesiol.* **2019**, *85*, 971–980.
10. Hodgson, C.L.; Hayes, K.; Everard, T.; Nichol, A.; Davies, A.R.; Bailey, M.J.; Tuxen, D.V.; Cooper, D.J.; Pellegrino, V. Long-Term Quality of Life in Patients with Acute Respiratory Distress Syndrome Requiring Extracorporeal Membrane Oxygenation for Refractory Hypoxaemia. *Crit. Care* **2012**, *16*, R202.
11. Mirabel, M.; Luyt, C.-E.; Leprince, P.; Trouillet, J.-L.; Léger, P.; Pavie, A.; Chastre, J.; Combes, A. Outcomes, Long-Term Quality of Life, and Psychologic Assessment of Fulminant Myocarditis Patients Rescued by Mechanical Circulatory Support. *Crit. Care Med.* **2011**, *39*, 1029–1035.
12. Muller, G.; Flecher, E.; Lebreton, G.; Luyt, C.-E.; Trouillet, J.-L.; Bréchot, N.; Schmidt, M.; Mastroianni, C.; Chastre, J.; Leprince, P.; et al. The ENCOURAGE Mortality Risk Score and Analysis of Long-Term Outcomes after VA-ECMO for Acute Myocardial Infarction with Cardiogenic Shock. *Intensive Care Med.* **2016**, *42*, 370–378.
13. Corsi, F.; Lebreton, G.; Bréchot, N.; Hekimian, G.; Nieszkowska, A.; Trouillet, J.-L.; Luyt, C.-E.; Leprince, P.; Chastre, J.; Combes, A.; et al. Life-Threatening Massive Pulmonary Embolism Rescued by Venoarterial-Extracorporeal Membrane Oxygenation. *Crit. Care* **2017**, *21*, 76.
14. Risnes, I.; Wagner, K.; Nome, T.; Sundet, K.; Jensen, J.; Hynås, I.A.; Ueland, T.; Pedersen, T.; Svennevig, J.L. Cerebral Outcome in Adult Patients Treated with Extracorporeal Membrane Oxygenation. *Ann. Thorac. Surg.* **2006**, *81*, 1401–1406.
15. Tramm, R.; Ilic, D.; Sheldrake, J.; Pellegrino, V.; Hodgson, C. Recovery, Risks, and Adverse Health Outcomes in Year 1 After Extracorporeal Membrane Oxygenation. *Am. J. Crit. Care* **2017**, *26*, 311–319.
16. Chen, K.-H.; Chen, Y.-T.; Yeh, S.-L.; Weng, L.-C.; Tsai, F.-C. Changes in Quality of Life and Health Status in Patients with Extracorporeal Life Support: A Prospective Longitudinal Study. *PLoS One* **2018**, *13*, e0196778.
